# Institutional capacity for health systems research in East and Central African schools of public health: knowledge translation and effective communication

**DOI:** 10.1186/1478-4505-12-20

**Published:** 2014-06-02

**Authors:** Richard Ayah, Nasreen Jessani, Eric M Mafuta

**Affiliations:** 1 School of Public Health, College of Health Sciences, University of Nairobi, P.O. BOX 19676-00202, KNH, Nairobi, Kenya; 2 Department of International Health, Johns Hopkins School of Public Health, 615 N. Wolfe St, Baltimore 21205, MD, USA; 3 School of Public Health, University of Kinshasa, P.O. BOX 11850, Kinshasa 1, Democratic Republic of the Congo

**Keywords:** Health systems research, Knowledge translation, Organizational capacity, Schools of public health, Sub-Saharan Africa

## Abstract

**Background:**

Local health systems research (HSR) provides policymakers and practitioners with contextual, evidence-based solutions to health problems. However, producers and users of HSR rarely understand the complexities of the context within which each operates, leading to the “know–do” gap. Universities are well placed to conduct knowledge translation (KT) integrating research production with uptake. The HEALTH Alliance Africa Hub, a consortium of seven schools of public health (SPHs) in East and Central Africa, was formed to build capacity in HSR. This paper presents information on the capacity of the various SPHs to conduct KT activities.

**Methods:**

In 2011, each member of the Africa Hub undertook an institutional HSR capacity assessment using a context-adapted and modified self-assessment tool. KT capacity was measured by several indicators including the presence of a KT strategy, an organizational structure to support KT activities, KT skills, and institutional links with stakeholders and media. Respondents rated their opinions on the various indicators using a 5-point Likert scale. Averages across all respondents for each school were calculated. Thereafter, each school held a results validation workshop.

**Results:**

A total of 123 respondents from all seven SPHs participated. Only one school had a clear KT strategy; more commonly, research was disseminated at scientific conferences and workshops. While most respondents perceived their SPH as having strong institutional ties with organizations interested in HSR as well as strong institutional leadership, the organizational structures required to support KT activities were absent. Furthermore, individual researchers indicated that they had little time or skills to conduct KT. Additionally, institutional and individual links with policymakers and media were reported as weak.

**Conclusions:**

Few SPHs in Africa have a clear KT strategy. Strengthening the weak KT capacity of the SPHs requires working with institutional leadership to develop KT strategies designed to guide organizational structure and development of networks with both the media and policymakers to improve research uptake.

## Background

Health systems research (HSR) can be broadly defined as the production of new knowledge to improve how societies organize themselves to achieve health goals [1]. As the field of HSR has gained ground, so has the importance of ensuring that the research products are responsive to policy priorities and, consequently, that policies are evidence informed. The utility of HSR derives directly from its ability to inform policy and decision-making. However, the producers and the users of research evidence rarely understand the complexities of the context within which each operates. This has raised concerns of the “know–do” gap – the gap between what is known and what is done in practice – and, consequently, the need to bridge it. These concerns have been voiced at global forums such as WHO’s “Bridging the ‘know–do’ gap” meeting [2] and the 2004 Ministerial Summit, where Ministers of Health and delegates called “*for national governments to establish sustainable programs to support evidence-based public health and health care delivery systems, and evidence-based health related policies*” [3]. These declarations spurred activity at the local, regional, and international levels [4-6].

Over time, a variety of strategies have emerged to bridge this gap, with the most comprehensive being the concept of knowledge translation (KT), synthesis, and exchange. While there are several interpretations of KT [7,8], fundamentally, it seeks to involve multiple actors and processes at multiple junctures to promote the research-policy paradigm. KT is defined by the Canadian Institutes of Health Research as “*a dynamic and iterative process that includes the synthesis, dissemination, exchange and ethically sound application of knowledge to improve health, provide more effective health services and products, and strengthen the health care system*” [9].

The last decade has witnessed much experimentation in terms of enhancing KT, including training individual researchers to communicate their research results in different ways for different audiences, convening national policy dialogues to promote interaction between decision-makers and researchers, creating dedicated KT platforms to serve as intermediaries between researchers and research users, and bringing the media on board to appreciate the implications of research results on the health of the public. Several organizations, such as the European Observatory on Health Systems and Policies and the Milbank Memorial Fund, play the role of knowledge brokers providing opportunities for researchers and policymakers to dialogue on key issues [10]. Others, such as the Health Evidence Network and the Genomics Forum, endeavor to package research results in a more digestible way for decision-makers. The evolution of KT platforms, such as the Regional East African Community Health Policy (REACH) Initiative [11], the Zambian Forum for Health Research (ZAMFOHR) [4], WHO’s Evidence Informed Policy Network (EVIPNet) [12], the European Union’s Support of Use in Research, and others, are a direct result of the need for an intermediary to facilitate the use of policy-relevant research and broad spectrum agreement on setting health research priorities. Funding agencies such as Canada’s International Development Research Centre (IDRC) and the UK Department for International Development (DFID) have increased their interest in the role that research institutions play in KT and in KT platforms as evidenced by the number of calls for requests for proposals.

KT platforms exist to create and nurture links among researchers, policymakers, and other research users. These links should draw the research and policy communities closer together to ultimately create cycles of policy-informed evidence and evidence-informed policy. Ideally, KT platforms are led by trustworthy, well-connected, and credible experts, and intermediaries who have excelled in various fields, including evidence gathering, critical appraisal, facilitation, communication, and networking.

Universities have frequently been regarded as key institutions in processes of social change and development. The most explicit role they have is the production of highly skilled labor and research output to meet perceived socioeconomic needs. Globally, there is an increased importance placed on knowledge, especially in securing a national competitive advantage [13]. This is echoed in development plans such as in Rwanda ‘Vision 2020’ to transform Rwanda’s economy into a middle-income country by transforming from a subsistence agriculture economy to a knowledge-based society [14]. Other countries in the region have adopted similar plans of the future, such as Kenya’s ‘Vision 2030’ [15], Tanzania’s ‘Vision 2025’ [16], and Uganda’s ‘Vision 2045’ [17].

Because universities have traditionally been recognized as knowledge producers, they are well placed to integrate research production with research uptake. Knowledge creation (i.e., primary research), knowledge distillation (i.e., the creation of systematic reviews and guidelines), and knowledge dissemination (i.e., appearances in journals and presentations) are not enough on their own to ensure the use of knowledge in decision-making or to guarantee the role of universities as one of the institutions charged with transforming society [13]. Knowledge production, accumulation, transfer, and application have become major factors in socio-economic development and are increasingly at the core of national development strategies [18]. Universities, as agents of change, therefore need to engage in KT for policymakers to effect evidence-based change that improves society. Context is an important element particularly for HSR, which looks at policies, organizations, and programs [19]. This awareness gives strong backing to the argument that, in order to strengthen health systems, partnerships between government and academia are imperative [10,20].

In Africa, institutes of higher education and, increasingly, networks such as the Association of African Universities are beginning to participate actively in development policymaking [21]. However, the capacity for African universities to engage in KT is not well documented. Understanding the capacity gaps and challenges and opportunities in enhancing these capacities served as the impetus for a capacity assessment across seven schools of public health in East and Central Africa.

The Higher Education Alliance for Leadership Through Health (HEALTH) formed in 2008 as a consortium of seven schools of public health (SPHs) in East and Central Africa: Jimma University College of Public Health and Medical Science (CPHMS, Ethiopia), Kinshasa School of Public Health (KSPH, Democratic Republic of the Congo (DRC)), Makerere School of Public Health (MakSPH, Uganda), Moi University School of Public Health (MUSOPH, Kenya), Muhimbili School of Public Health and Social Sciences (MUSPHSS, Tanzania), National University of Rwanda School of Public Health (NURSPH, Rwanda), and University of Nairobi School of Public Health (SPHUoN, Kenya). Although each of the SPHs participating in the HEALTH Alliance has an interest in HSR, the capacity of each SPH to conduct HSR and promote its uptake varies substantially. The HEALTH Alliance was formed out of a need to have a coordinated effort to strengthen HSR and encourage shared learning. Realizing that they had similar objectives, the Future Health Systems (FHS) consortium [22] and the HEALTH Alliance came together in 2011 to form the Africa Hub. The Africa Hub’s membership comprises the same SPHs that make up the HEALTH Alliance. Specifically, the Africa Hub aims to i) assess and strengthen capacity for HSR in the SPHs, ii) extend networks for communicating learning in HSR and facilitate an exchange of ideas and research among countries, and iii) improve capacity to communicate and promote uptake of research evidence in policy and decision-making. To date, the Africa Hub has been supported by the FHS consortium.

This paper is based on an HSR capacity self-assessment that was conducted by the seven SPHs, with the objectives of ascertaining existing capacities for HSR; building consensus around HSR capacity development strategies for each SPH; and making an initial and rapid assessment of HSR priorities in the different countries involved in the HEALTH Alliance. This paper, which is one in a series of four [23-25], presents findings on the experience and perceptions of participants on the capacity of these seven SPHs in East and Central Africa to carry out KT and communication activities.

## Methods

In order to better understand the motivation, challenges, and capacity of the seven SPHs to engage in KT, all seven schools participated in an organizational self-assessment in 2011. A common protocol, derived from an instrument that IDRC uses to assess the organizational capacity needs of its partner research organizations [26], was adapted and then refined to focus on HSR. The initial protocol was shared and revised by representatives from the various SPHs at a workshop held in Uganda in June 2011. Amendments and clarifications were made to ensure relevance of the tool. Each school obtained ethical approval for the study from their local ethics committee prior to the start of data collection (one exception to this was at MUSPHSS, Tanzania, where the assessment was regarded as part of an ongoing routine capacity strengthening effort). The assessment had three parts: a self-assessment, a review of internal documents in order to generate a profile of HSR in the institution, and key informant interviews of internal and external stakeholders. More details on the refinement of the tool as well as a final version of the HSR assessment tool is reported elsewhere [25]. Only the self-assessment had direct questions related to KT.

Data collection in each school was led by a staff member involved in teaching health systems, who was appointed by the Dean to be the focal person for the HSR assessment process. The assessment was carried out in three steps. The focal person identified key persons including the deans, deputy deans, heads of departments, and staff within the institution. Inclusion criteria were those who taught, did research, or had a stake in health systems. Further, to have a common understanding of HSR, the questionnaire began by providing a definition of HSR and offering examples of studies we believe reflect HSR as well as those that do not. Each of the identified individuals was asked to complete the self-assessment questionnaire on their own. The self-assessment supported the exploration of a number of aspects of organizational HSR capacity. However, the results relevant to capacity for KT were drawn from section two: “*Capacity development and collaborative research in health systems*”. Within this section, faculty currently or potentially engaged in HSR were asked to complete a subjective self-administered questionnaire that sought their opinion on HSR capacity at their organization. Out of 26 questions, 13 focused directly on KT. In assessing the capacity of the individual schools to conduct KT, we looked at several parameters, namely the presence of a KT strategy, an organizational structure to support KT activities, KT skills, capacity to engage in results dissemination, institutional links with stakeholders, and media linkages. Respondents rated their opinions using a 5-point Likert scale (1 = strongly disagree, 5 = strongly agree) to indicate the extent to which they agreed with each statement. For each statement, the scores obtained at a school were summed and divided by the number of respondents to give an average score, as described elsewhere [23]. Each SPH collated the results from their assessments and documented their capacity strengths, challenges, and potential solutions.

In addition, the focal person collected basic relevant data, reviewing documents, such as strategic plans and annual reports, to complete a short HSR profile questionnaire on the status of HSR within each school. Data was collected on paper surveys, then transferred and analyzed using excel. Thereafter, individual schools held dissemination workshops to discuss and validate the results of their capacity assessments. These were then shared and discussed among all Africa Hub member teams in December 2011. The results below provide an overview of identified gaps in KT and communication, recognized across all seven schools as well as those unique to each context.

## Results

The results are organized around a framework for evaluating KT capacity in terms of the organizational policies that should define the structure and operations, leadership and individuals with the necessary skills, and available networks to build and sustain continuity. A total of 123 respondents across the seven SPHs answered the self-assessment. The response rate varied from 9% in CPHMS, Ethiopia, to 92% in KSPH, DRC (Table 1). This variation in response rate may be a function of the proportion of faculty interested in HSR and in the size of the faculty. In the latter case, CPHMS, Ethiopia, had 285 academic staff while SPHUoN, Kenya had the fewest, with only 18 faculty members.

**Table 1:** Number of faculty and study participants by school

**School**	**Year established**	**No. of faculty**	**Study participants**	**Response rate**
Makerere School of Public Health, Uganda	1974	58	15	26%
Kinshasa School of Public Health, DRC	1985	38	35	92%
Muhimbili School of Public Health, Tanzania	1991	43	16	37%
Moi University School of Public Health, Kenya	1997	35	22	63%
National University of Rwanda School of Public Health, Rwanda	2000	19	4	21%
Jimma University College of Public Health and Medical Science, Ethiopia	2009	285	26	9%
University of Nairobi School of Public Health, Kenya	2010	18	5	28%

While each of the schools had a strategic plan, only one out of seven schools, MakSPH, Uganda, had KT as an explicit item in their strategic plan. Their strategy advocated for knowledge generation and transfer through various outreach activities with emphasis on having personnel and mechanisms for KT to drive the creation of relevant policies. A more common approach, seen in the strategic documents from four of the seven schools, was the dissemination of research findings at workshops and conferences. This may reflect the traditional belief that researchers interpret their roles as primarily to generate knowledge and seldom to rearticulate it for various purposes.

The extent to which research in HSR is supported and subsequently utilized by decision-makers often depends on whether they have a basic understanding and interest in HSR results. We reviewed three aspects of organizational structure in the self-assessment: leadership support for HSR – the extent to which leaders provide needed resources; the ability of dedicated communication staff to support KT of HSR; and the capacity of faculty to engage in results dissemination. There was no consensus across the SPHs that there were individuals who could provide high-level leadership for HSR within their institutions (Table 2). SPHUoN, Kenya, scored 2.3, the lowest on this question; and NURSPH, Rwanda, scored 3, while the other schools scored 4.0 and above. These results are to some extent inconsistent with the perception of faculty that external stakeholders, such as Ministries of Health, had high levels of interest in HSR. On that question, scores ranged from 4.2 at MakSPH, Uganda, to 3.4 at MUSPHSS, Tanzania, indicating that there is, perhaps, an untapped possibility of greater institutional engagement with policymakers if there is greater commitment by SPH leadership in supporting KT activities as results in Table 2 indicate.

**Table 2:** Perceived interest in health systems research across the schools of public health (SPHs)

	**KSPH**	**MakSPH**	**MUSPHSS**	**SPHUoN**	**MUSPH**	**CPHMS**	**NURSPH**
**Number of respondents per SPH**	**n = 35**	**n = 15**	**n = 16**	**n = 5**	**n = 22**	**n = 26**	**n = 4**
I feel confident that there are individuals in this college who can provide high level leadership for HSR.	4.2	4.2	4.2	2.3	4.4	4.0	3
Key institutions in this country, such at the Ministry of Health, have a strong interest in HSR.	3.7	4.2	3.4	4.0	3.9	3.9	4.0

However, when asked whether the SPH’s communication staff had the capacity to “effectively communicate HSR findings to many different audiences,” the average scores across the schools were relatively low, ranging from 3.9 (KSPH, DRC) to 1.7 (SPHUoN, Kenya) (Table 3). Table 3 also shows that individual interaction and communication with decision-makers and policymakers was not perceived to be extensive at any SPH (range 2.3–3.8). Evidence dissemination often depends on relationships as well as windows of opportunity. When respondents were asked about their perceptions on individual interactions and communications with decision-makers, most SPHs scored at or above 3.0 with KSPH, DRC, responding very positively with a score of 3.8, the other schools ranging from 3.7 to 3.0 with SPHUoN, Kenya, the outlier at 2.3. The final question “This school has a strong communications staff and capacity to effectively communicate HSR findings to many different audiences” elicited, once again, a positive reflection from KSPH, DRC, at 3.8. In general, however, this score was relatively low with scores ranging from 3.4 (MakSPH, Uganda) to 1.7 (SPHUoN, Kenya).

**Table 3:** Capacity of schools of public health (SPHs) to disseminate health systems research results

	**KSPH**	**MakSPH**	**MUSPHSS**	**SPHUoN**	**MUSPH**	**CPHMS**	**NURSPH**
**Number of respondents per SPH**	**n = 35**	**n = 15**	**n = 16**	**n = 5**	**n = 22**	**n = 26**	**n = 4**
Researchers at this college have the **time to disseminate** their findings to policy makers through knowledge translation mechanisms such as policy briefs or policy dialogues or one-on-one discussions, etc.	3.3	3.7	3.3	2.3	3.5	3.3	3.0
Researchers at this college have the **motivation to disseminate** their findings to policy makers through knowledge translation mechanisms such as policy briefs or policy dialogues or one-on-one discussions, etc.	3.4	3.8	2.8	2.0	3.3	3.1	3.5
Researchers at this college have the **skills to disseminate** their findings to policy makers through knowledge translation mechanisms such as policy briefs or policy dialogues or one-on-one discussions.	3.9	3.6	3.2	2.0	3.5	3.1	3.0
This SPH has capacity in the area of individual interaction and communication with decision makers/policy makers?	3.8	3.7	3.1	2.3	3.6	3.0	3.0
This school has a strong communications staff and capacity to effectively communicate HSR findings to many different audiences.	3.9	3.4	2.9	1.7	2.6	3.2	2.0

On the self-assessment, there were three other questions on the capacity to disseminate research results, which explored specific determinants that have been previously documented in the literature as well as through anecdotal discussions: individual researchers having the time, personal motivation, and the skills to share their findings with policymakers through various KT mechanisms (Table 3). SPHs scored similarly across the three dimensions within their schools, but average scores varied across schools. For instance, CPHMS, Ethiopia, ranked itself at 3.3, 3.1, and 3.1, respectively, while SPHUoN, Kenya, ranked itself at 2.3, 2, and 2.

There is an increasing emphasis on the utilization of research results by a variety of people beyond academia. Each of the SPHs surveyed produces knowledge in various forms, primarily as student theses and faculty publications. Despite that, when asked whether Ministry of Health (MOH) officials and staff at health facilities value and use the evidence that each SPH provides, most SPHs scored themselves at 3.0 or above for both. However, NURSPH, Rwanda, and MUSPHSS, Tanzania, scored low (2.0) for research use by health facilities while MUSOPH, Kenya, and CPHMS, Ethiopia, both felt that health facilities utilized their HSR more than MOH officials did (Table 4).

**Table 4:** Perception of research uptake by stakeholders

	**KSPH**	**MakSPH**	**MUSPHSS**	**SPHUoN**	**MUSPH**	**CPHMS**	**NURSPH**
**Number of respondents per school of public health**	**n = 35**	**n = 15**	**n = 16**	**n = 5**	**n = 22**	**n = 26**	**n = 4**
Ministry of Health officials value the evidence that we provide and draw upon it in their work.	3.7	3.5	2.2	3.0	3.0	3.2	4.0
Health facilities and health staff value the evidence that we provide and draw upon it in their work.	3.4	3.2	2.0	3.0	3.5	3.3	2.0

Research uptake, as discussed in the previous paragraph, can be enhanced through good institutional and media linkages. The self-assessment included queries about six types of institutional linkages: MOH, health facilities and health staff, organizations engaged in HSR nationally, organizations engaged in HSR internationally, media, and NGOs. Respondents generally agreed that the various schools had strong links to organizations interested in HSR (Table 5). MakSPH, Uganda, and KSPH, DRC, scored high (>3.0) across all dimensions. NURSPH, Rwanda, respondents were extremely content with its links to MOH (5.0) and were happy in general with links to all other organizations mentioned above except for health facility linkages (2.0) and the media (1.3). Other than MakSPH, Uganda, which reported a score of 3.7, schools had low scores (range 3.0–1.3) when asked whether they had strong institutional linkages to media organizations. Ties to other organizations engaged in HSR within as well as outside of the country were reported to be relatively strong (range 4.0–2.7).

**Table 5:** Schools of public health (SPH) institutional links

	**KSPH**	**MakSPH**	**MUSPHSS**	**SPHUoN**	**MUSPH**	**CPHMS**	**NURSPH**
**Number of respondents per SPH**	**n = 35**	**n = 15**	**n = 16**	**n = 5**	**n = 22**	**n = 26**	**n = 4**
This SPH has strong institutional links to the MOH/units within the MOH with an interest in HSR.	3.7	3.9	2.4	3.0	3.8	3.2	5.0
This SPH has strong institutional linkages to health facilities and health staff who can be engaged in HSR.	3.6	3.8	1.9	3.0	3.8	3.4	2.0
This SPH has strong institutional linkages to other organizations engaged in HSR in **our** country.	3.9	3.7	2.7	3.0	3.6	3.4	4.0
This SPH has strong institutional linkages to other organizations engaged in HSR in **other** countries.	3.3	4.0	3.2	3.0	3.6	3.5	4.0
This SPH has strong institutional linkages to media organizations.	3.0	3.7	2.0	2.7	1.9	2.9	1.3
This SPH has strong institutional linkages to NGOs in our country which are interested in health systems.	3.7	3.5	2.3	2.7	3.5	3.4	4.0

## Discussion

We set out to appraise the capacity of selected SPHs in East and Central Africa to carry out KT and communication of their HSR. This was part of a broader assessment of the schools’ capacity to design, implement, and monitor HSR at country and regional levels. Capacity in this context can be defined as the ability of each SPH to meet its stated goals in an efficient and effective manner. In evaluating capacity, four core issues must be considered [23]: Do the institutional arrangements permit the vision, mission, and strategy of the organization to be realized? Is there strong leadership and governance? Are the necessary knowledge and skills available within the school, university, and government? Are there accountability mechanisms and do they incorporate strategic relationships? Only one school out of the seven SPHs reported having a formal strategy of KT engagement, indicating possible weaknesses in the institutional arrangements in the other six schools. Despite the absence of clear strategies for KT, five out of the seven schools reported having confidence that there was high-level leadership available for HSR in their institution and in the government. However, this belief was not sufficient to motivate researchers to disseminate their findings. This disconnect may reflect the traditional idea that academic and political leaders view universities as primarily responsible for the production of highly skilled labor and research [13]. Thus, the obstacle blocking effective KT and communication of HSR in the SPHs may be a lack of awareness of KT and, therefore, of how to structure the organization to conduct KT.

To successfully engage in creating and translating research, it is vital for organizations to cultivate contacts with other institutions and organizations. Formal links among individuals and institutions promotes a healthy exchange of approaches and resources [26]. KT requires that schools have the capacity to define their research questions and disseminate their results. For HSR, it is important that the framework in which researchers operate include targeting policymaking [27]. Defining research questions and methodologies is the first step in the research cycle [28]. This requires that schools have the resources and individuals within the schools have the skills, motivation, time, and credibility to transfer research knowledge. The entire KT process is skill-intensive and time-consuming [29]. When considering whether, at their institution, faculty had the time and the skills to disseminate their findings through various KT mechanisms, the average responses among respondents at all of the SPHs fell into the category, “neither agree nor disagree”. This may be because, at each SPH, only a small pool of senior staff have the necessary networks and skills to perform KT effectively, but these individuals already carry a heavy burden including fund-raising, mentoring new researchers, and overseeing small grants. This leaves them with limited time to specialize and develop their own skills fully [30].

Effective and sustainable KT may benefit from the development of organizational knowledge infrastructures [31]. Ellen et al. developed a framework that identified potential organizational components that a healthcare system could have in its research knowledge infrastructure [32]. Among them were activities used to link research to action, including push efforts (i.e., efforts undertaken by researchers to disseminate research evidence to knowledge users), pull efforts (i.e., efforts by knowledge users to access and use research evidence), and exchange efforts. The lack of organizational structure geared towards KT within the seven SPHs explains the perceived weaknesses of the SPHs in effectively communicating HSR findings.

Despite the weak KT infrastructure, the SPHs reported having strong institutional links with organizations interested in HSR, especially MOHs and, to a lesser extent, non-governmental organizations. Oftentimes, such links arise through networks created with graduates of SPHs who subsequently accept positions within the MOHs and various NGOs. Further research would help identify whether such links are reliant on individual relations or whether they manifest more broadly as institutional relationships. Individual relations may be seen as a facilitator rather than a barrier, because a few individuals with strong connections to policymakers and funders may be a more valuable strategy of KT than relying on a barrage of information for policymakers to digest [33]. If academic faculty felt that these linkages were important, it would be incumbent upon SPHs to explore strategies for addressing the said reasons.

Mass media campaigns have long been tools used for promoting public health [34]. However, it is uncommon amongst academia and, therefore, not surprising that the seven SPHs reported having weak links with media. Six of the seven schools reported low scores in terms of their institutional linkages to media, and most were either unsure or disagreed that they had the capacity to effectively communicate to diverse audiences. Part of this disconnect may be the reality that the print media in many countries does not report much on evidence-based HSR [35], and that the role of mass media is symbolic, used primarily to legitimize and sustain predetermined positions [28]. There is also insufficient evidence on the effectiveness of the use of mass media to change the behavior of health professionals in developing countries [36]. However, a growing body of evidence suggests that constructive discussion on social media and the resultant public visibility can be beneficial for scientists. Further, such media can impact research by creating an online scientific network bringing together researchers and policymakers [37]. One fear that SPHs may have at both institutional and individual levels is the way in which media can distort scientific information. Negative press can have a damaging effect on careers and even science itself [38]. In addition, it is important to understand the role of media in each of the countries and the degree of freedom that the press has; Uganda, DRC, Rwanda, and Ethiopia score relatively poorly in terms of press freedom [39]. Given the possible conflicting roles that media can play, a careful proactive media-engagement strategy should be developed by each of the schools before they engage in KT. The communication strategy must not only inform the audience but also capture their attention and inspire them to action [40].

Our findings are not unusual; in a survey of low- and middle-income countries, Lavis et al. found that KT activities, such as targeted dissemination of research products and the development of the capacity of target audiences to find and use research, were rarely undertaken [41]. However, for specific areas of research, such as diarrheal disease, activity was higher than in other areas, such as malaria prevention, irrespective of economic development [41]. This should be seen as an encouraging sign for health systems researchers.

### Study limitations

The primary data collection tool was a self-assessment questionnaire; therefore, individuals may have interpreted terms like “capacity”, differently. Given the small sample size at each participating SPH we could not assess rigorously the validity or reliability of the tool, but future studies with larger samples could usefully contribute to this. However, given the small number of faculty involved in HSR, the effect of selection bias is likely limited and the sampling reflected the need to grow HSR. While we sought to apply the common lessons learned across the seven different SPHs, it is important to recognize the different contexts within which they operate. This may reflect the generally low scores obtained from SPHUoN, Kenya, when compared with MakSPH, Uganda, especially in terms of leadership and institutional frameworks set to advance KT.

## Conclusions and recommendations

The context specificity of health policy and systems research constitutes a major challenge and requires researchers to work closely with policymakers to ensure that research improves health outcomes. African SPHs have traditionally focused on training as their main output. Given the increasing importance of HSR, SPHs are well placed to integrate research production with research uptake and become KT platforms. Our study shows, however, that among the seven SPHs, there is weak capacity to translate knowledge. Strategies to improve this capacity should include working with institutional leadership to develop clear KT strategies, including the development of institutional networks and media engagement, which together would provide the organizational support needed to improve research uptake.

## Abbreviations

CPHMS: College of Public Health and Medical Sciences, Jimma University, Ethiopia; DRC: Democratic Republic of the Congo; HEALTH: Higher Education Alliance for Leadership Through Health; HSR: Health systems research; IDRC: International Development Research Centre; KSPH: Kinshasa School of Public Health, Democratic Republic of the Congo; KT: Knowledge translation; MOH: Ministry of Health; MUSPHSS: Muhimbili University of Health and Allied Sciences, School of Public Health, Tanzania; MUSPH: Moi University, School of Public Health, Kenya; MakSPH: Makerere University College of Health Sciences, Uganda; NURSPH: National University of Rwanda School of Public Health, Rwanda; SPH: School of public health; SPHUoN: University of Nairobi School of Public Health, Kenya.

## Competing interests

The authors declare that they have no competing interests.

## Authors’ contributions

All three authors were involved in the design the assessment tool. RA and EM were involved in the implementation in their respective SPHs. RA and NJ wrote first drafts of the manuscript. EM provided substantial input as well as reviewing the draft manuscript. All of the authors read and approved the final manuscript.
